# Risk-Stratifying Pituitary Adenoma Treatment: A Cohort Analysis and Risk Prediction of Hypopituitarism

**DOI:** 10.3390/jcm14186656

**Published:** 2025-09-22

**Authors:** Adnan Agha, Shriram Dorairaj Gunasekaran, Entessor Mohammed Noor, Khaled Mohammed Asad Al Dahmani

**Affiliations:** 1 Department of Internal Medicine, College of Medicine and Health Sciences, United Arab Emirates University, Al Ain P.O. Box 15551, United Arab Emirates; 2 Department of Internal Medicine, Tawam Hospital, Al Ain P.O. Box 15258, United Arab Emirates; 3 Division of Endocrinology, Department of Internal Medicine, Tawam Hospital, Al Ain P.O. Box 15258, United Arab Emirates

**Keywords:** pituitary adenoma, hypopituitarism, risk prediction, prolactinoma, transsphenoidal surgery, radiotherapy

## Abstract

**Background/Objectives**: The management of pituitary adenomas involves balancing treatment efficacy with the risk of long-term morbidity, particularly treatment-induced hypopituitarism. While risk factors are qualitatively recognized, quantitative, individualized risk prediction tools for clinical practice are lacking. This study aims to evaluate and characterize the clinical features, hormonal profiles, and treatment outcomes of pituitary adenomas, and to develop and validate a pragmatic clinical prediction model for new-onset hypopituitarism. **Methods**: We conducted a retrospective cohort study of 215 patients diagnosed with pituitary adenomas, selected from 647 sellar lesions screened at a tertiary referral center between January 2010 and December 2020. Primary outcomes included adenoma size control, hormonal remission in functioning adenomas, and the development of new-onset hypopituitarism. A multivariable logistic regression model was developed to identify independent predictors of new-onset hypopituitarism, and its performance was assessed for discrimination and calibration. **Results**: The cohort consisted of 107 prolactinomas (49.8%), 77 non-functioning adenomas (35.8%), 18 GH-secreting (8.4%), and 8 ACTH-secreting (3.7%) adenomas, with a mean age of 43.2 ± 14.1 years and a female predominance (59.1%). At a median follow-up of 4.8 years, overall adenoma control was 92.1%. Radiotherapy achieved 100% adenoma control but was associated with the highest incidence of new hypopituitarism at 5 years (34.3%), significantly greater than medical therapy (5.6%, *p* < 0.001) and surgery (13.0%, *p* < 0.01). The final risk prediction model, incorporating treatment modality, baseline hypopituitarism, macroadenoma, age >50 years, and cavernous sinus invasion, demonstrated good discrimination (C-statistic = 0.82; 95% CI: 0.76–0.88) and excellent calibration (Hosmer–Lemeshow *p* = 0.42). **Conclusions**: Treatment modalities for pituitary adenomas have distinct risk–benefit profiles. Our validated, points-based risk model provides a transparent and clinically applicable tool to quantify an individual patient’s risk of developing hypopituitarism. This model can be integrated into clinical practice to facilitate shared decision-making and guide personalized surveillance strategies.

## 1. Introduction

Pituitary adenomas, now classified as pituitary neuroendocrine tumors (PitNETs) under the 2022 World Health Organization classification, are a heterogeneous group of neoplasms that account for 10–15% of all intracranial tumors [1]. With a prevalence estimated between 78 and 94 per 100,000 population, their clinical significance extends beyond mass effects to include a wide spectrum of endocrine manifestations, from hormone hypersecretion syndromes to debilitating hypopituitarism [2,3,4]. Although overwhelmingly benign, these adenomas can cause significant morbidity through hormonal dysfunction, visual compromise, and treatment-related complications [5]. Despite a prevalence approaching 20% in autopsy and radiological studies, only a fraction of pituitary adenomas become clinically significant, highlighting the need to optimize treatment strategies that balance tumor control with minimizing harm [6].

The management of pituitary adenomas has evolved into a sophisticated, multimodal paradigm tailored to adenoma subtype, size, hormonal activity, and patient-specific factors [5,7]. Medical therapy, particularly with dopamine agonists (DAs), remains the cornerstone of prolactinoma management, as reaffirmed by the 2023 Pituitary Society international consensus statement, which notes high rates of biochemical control and tumor shrinkage [7]. However, the prospect of lifelong therapy and variable remission rates after DA withdrawal present ongoing clinical challenges [5,7]. For most other adenoma subtypes, transsphenoidal surgery (TSS) is the primary treatment, aiming for maximal safe resection to relieve mass effect and achieve hormonal remission [5]. The field has seen a significant shift towards endoscopic techniques, which offer superior panoramic visualization; recent meta-analyses suggest that while gross total resection rates are comparable to traditional microscopic approaches, endoscopy may be associated with improved endocrine outcomes and lower rates of certain complications [8]. Radiotherapy, particularly stereotactic radiosurgery (SRS), serves as a critical adjuvant or salvage therapy for residual or recurrent disease, with recent systematic reviews from 2024 demonstrating excellent long-term tumor control rates exceeding 90% for non-functioning pituitary adenomas [9,10].

However, each treatment modality carries its own specific risks, particularly related to pituitary hormonal function. Partial or panhypopituitarism represents a major long-term complication, affecting quality of life and mortality [11,12]. The incidence of pituitary hormonal deficiencies varies significantly by treatment modality, generally ranging from 5 to 20% following medical therapy, 10 to 30% after surgery, and 20 to 80% following radiotherapy [13,14]. Recent meta-analyses confirm that radiation-induced hypopituitarism is common in adults, with a pooled prevalence approaching 50% in some cohorts, and SRS-specific studies report new hypopituitarism rates of 13–21% [13,15]. This significant endocrine cost underscores the critical need to balance the goal of adenoma control with the preservation of pituitary function. While clinicians are qualitatively aware of risk factors such as radiotherapy exposure, large tumor size, and pre-existing pituitary dysfunction, a significant gap exists in the ability to translate this knowledge into a quantitative, individualized risk estimate at the point of care. The recent emergence of complex machine learning (ML) and artificial intelligence (AI) models for predicting pituitary adenoma recurrence and treatment outcomes highlights the trend towards data-driven prognostication [16]. However, many of these models function as “black boxes”, limiting their clinical interpretability and immediate applicability. There remains an unmet need for a transparent, validated, and easy-to-use clinical tool that can integrate key patient and tumor characteristics to provide a pragmatic risk score. Such a tool would empower clinicians and patients in the shared decision-making process, transforming a general understanding of risk into a personalized, actionable data point. This emphasis on a transparent, pragmatic tool distinguishes our approach from less interpretable "black box" models.

This study aimed to address these gaps by pursuing the following three primary objectives: (1) to evaluate the clinical characteristics and treatment outcomes of a large, unselected, contemporary cohort of patients with pituitary adenomas from the Middle East, a region underrepresented in the literature; (2) to quantify the incidence and identify independent predictors of treatment-related hypopituitarism across all therapeutic modalities; and (3) to develop and internally validate a pragmatic, points-based clinical risk score to predict this crucial long-term outcome.

## 2. Patients and Methods

### 2.1. Study Design and Population

This retrospective cohort study included all patients with sellar lesions (including pituitary adenomas) who were evaluated at Tawam Hospital, Al-Ain, United Arab Emirates, between January 2010 and December 2020. The study protocol was approved by the institutional review board, Tawam Human Research Ethics Committee (approval number MF2058-2023-946), and a waiver of informed consent granted due to the retrospective nature of the analysis of de-identified data.

**Figure 1:**
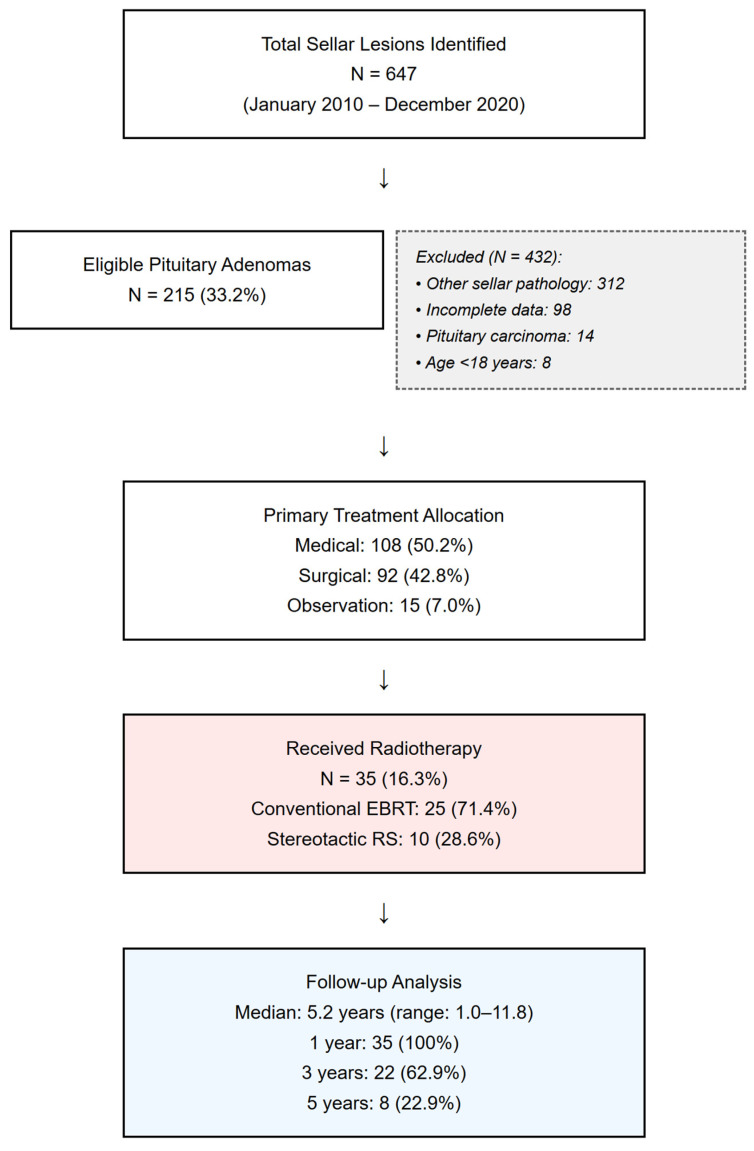
**STROBE flow diagram of patient selection.** Flow diagram illustrating the screening of 647 sellar lesions and the selection process, resulting in the final study cohort of 215 patients with pituitary adenomas.

From an initial screening of 647 consecutive patients with sellar lesions, we identified 215 patients who met the inclusion criteria, as detailed in the patient flow diagram (Figure 1).

### 2.2. Patient Selection Criteria

Patients were eligible for inclusion if they had a histologically confirmed or radiologically diagnosed pituitary adenoma, were 18 years of age or older at diagnosis, and had a complete baseline clinical and hormonal assessment. A minimum of 12 months of follow-up with available pre- and post-treatment hormonal data was also required.

Patients were excluded for the following reasons: diagnosis of other sellar pathologies, such as craniopharyngioma, Rathke’s cleft cyst, or meningioma (*n* = 312); incomplete data, including missing baseline hormones or imaging (*n* = 98); a diagnosis of pituitary carcinoma or metastases (*n* = 14); or an age younger than 18 years (*n* = 8).

### 2.3. Clinical and Hormonal Assessment

All patients underwent standardized evaluation per published guidelines [17]. This included a complete anterior pituitary hormone panel per Endocrine Society guidelines, including serum Prolactin (PRL), Thyroxine (T4), thyroid-stimulating hormone (TSH), growth hormone (GH), Insulin-like Growth Factor-1 (IGF-1), adrenocorticotropic hormone (ACTH), cortisol, testosterone/estradiol, follicle-stimulating hormone (FSH), and luteinizing hormone (LH). Dynamic pituitary function testing was performed where indicated. This included growth hormone (GH) suppression testing using a 75 g oral glucose tolerance test (OGTT), the low-dose dexamethasone suppression test (LDDST), and the insulin tolerance test (ITT) or short synacthen test for assessment of the adrenal axis [17]. Patients with hormonal excess syndromes like Cushing’s disease or Acromegaly, were diagnosed per consensus criteria, while hypopituitarism for each axis was defined by standard criteria per published guidelines [17,18,19].

### 2.4. Imaging Assessment

All patients underwent a standardized imaging assessment, which included contrast-enhanced pituitary magnetic resonance imaging (MRI) performed with a dedicated sellar protocol. Adenoma dimensions were systematically measured in three orthogonal planes. To classify the extent of adenoma invasion and extension, the Knosp grade was used to evaluate cavernous sinus involvement, and the Hardy–Wilson classification was applied to grade suprasellar extension.

### 2.5. Treatment Protocols

Treatment decisions were made by a multidisciplinary team. Surgical intervention, primarily via an endoscopic transsphenoidal approach, was indicated for patients with mass effect (e.g., visual field compromise), pituitary apoplexy, non-functioning macroadenomas, and most functioning adenomas, with the exception of prolactinomas. Surgery was also recommended for drug-resistant prolactinomas [7,17].

Radiotherapy was recommended as an adjuvant or salvage treatment for patients with documented growth of a residual adenoma (>20% increase in size), persistent hormone hypersecretion despite maximal medical and/or surgical therapy, or recurrent disease. The choice between conventional fractionated external beam radiotherapy (EBRT) and stereotactic radiosurgery (SRS) was made according to established paradigms. EBRT was typically delivered to a total dose of 45–50.4 Gy in 1.8 Gy daily fractions. SRS was delivered as a single fraction with a median margin dose of 14 Gy (range 12–18 Gy) [20,21].

Medical therapy regimens were based on international guidelines [17]. This included weekly cabergoline or daily bromocriptine for prolactinomas; somatostatin receptor ligands and/or pegvisomant for acromegaly; and steroidogenesis inhibitors (e.g., ketoconazole and metyrapone) or pasireotide for Cushing’s disease [18,19].

### 2.6. Outcome Measures and Definitions

The primary outcomes were as follows: (1) control of pituitary adenoma size, defined as no radiographic progression (>20% increase in maximum diameter); (2) hormonal remission for functioning adenomas, defined by standard criteria (e.g., prolactin normalization for prolactinomas; IGF-1 normalization and/or nadir GH < 1.0 µg/L on OGTT for acromegaly); and (3) the development of new pituitary hormone deficiency (hypopituitarism) in at least one axis after the initiation of treatment. Secondary outcomes included visual field improvement, requirement for hormone replacement therapy, and treatment-related complications.

### 2.7. Statistical Analysis

Statistical analysis was performed using SPSS version 28.0 (IBM Corp., Armonk, NY, USA). For descriptive statistics, normally distributed continuous variables are presented as mean ± standard deviation (SD), while non-normally distributed variables are presented as median and interquartile range (IQR). Categorical variables are presented as counts and percentages (*n*, %). Group comparisons were performed using the Student’s *t*-test or Mann–Whitney U test for continuous variables, and the chi-square or Fisher’s exact test for categorical variables. Time-to-event outcomes were evaluated using Kaplan–Meier analysis.

A multivariable logistic regression model was developed to predict the primary outcome of new-onset hypopituitarism. Variables with a *p*-value of < 0.10 in univariable analysis were considered for inclusion in the multivariable model, which was built using a backward stepwise selection method. Model performance was assessed for discrimination using the C-statistic (area under the receiver operating characteristic curve) and for calibration using the Hosmer–Lemeshow goodness-of-fit test. The final multivariable model was converted into a points-based risk score for clinical application. A two-tailed *p*-value of <0.05 was considered statistically significant. *p*-values for baseline comparisons in Table 1 are presented for exploratory purposes and were not adjusted for multiple comparisons. The multivariable logistic regression model was the primary analysis for identifying independent predictors, inherently controlling for the included covariates.

## 3. Results

### 3.1. Patient Demographics and Baseline Characteristics

The final cohort included 215 patients with a mean age of 43.2 ± 14.1 years and female predominance (127/215, 59.1%). The most common adenoma subtype was prolactinoma (107/215, 49.8%), followed by non-functioning adenoma (77/215, 35.8%), GH-secreting adenoma (18/215, 8.4%), ACTH-secreting adenoma (8/215, 3.7%), and other/multiple types (5/215, 2.3%). Macroadenomas (>10 mm) comprised 54.4% of the cohort (117/215), with a median maximum diameter of 18.5 mm (IQR 12–28 mm). Cavernous sinus invasion (Knosp grade ≥3) was present in 20.9% (45/215) and suprasellar extension in 41.4% (89/215) of patients.

At diagnosis, 33.0% of patients (71/215) had at least one pituitary hormone deficiency. The most common deficiency was gonadotropin (LH/FSH) deficiency (20.0%), followed by GH deficiency (14.9%), TSH deficiency (13.0%), and ACTH deficiency (9.8%). Multiple hormone deficiencies were present in 14.4% of patients (31/215). Hypopituitarism was three times more frequent in patients with macroadenomas as compared to those with microadenomas (48.7% vs. 15.3%, *p* < 0.001), and in those with invasive adenomas (56.5% vs. 23.4%, *p* < 0.001).

Medical therapy was the first-line treatment for 50.2% of patients (108/215), primarily for prolactinomas (89/108, 82.4%). Surgery was the initial treatment in 42.8% of patients (92/215), with an endoscopic approach used in 76.1%. Radiotherapy was administered to 16.3% (35/215) of the cohort, either as an adjuvant or salvage treatment; this included 25 patients who received EBRT and 10 who received SRS. A strategy of observation was chosen for 7.0% (15/215) of patients, who typically had small, asymptomatic, non-functioning adenomas. Baseline characteristics, stratified by receipt of radiotherapy, are detailed in Table 1.

### 3.2. Long-Term Outcomes Across All Treatment Modalities

**Figure 2:**
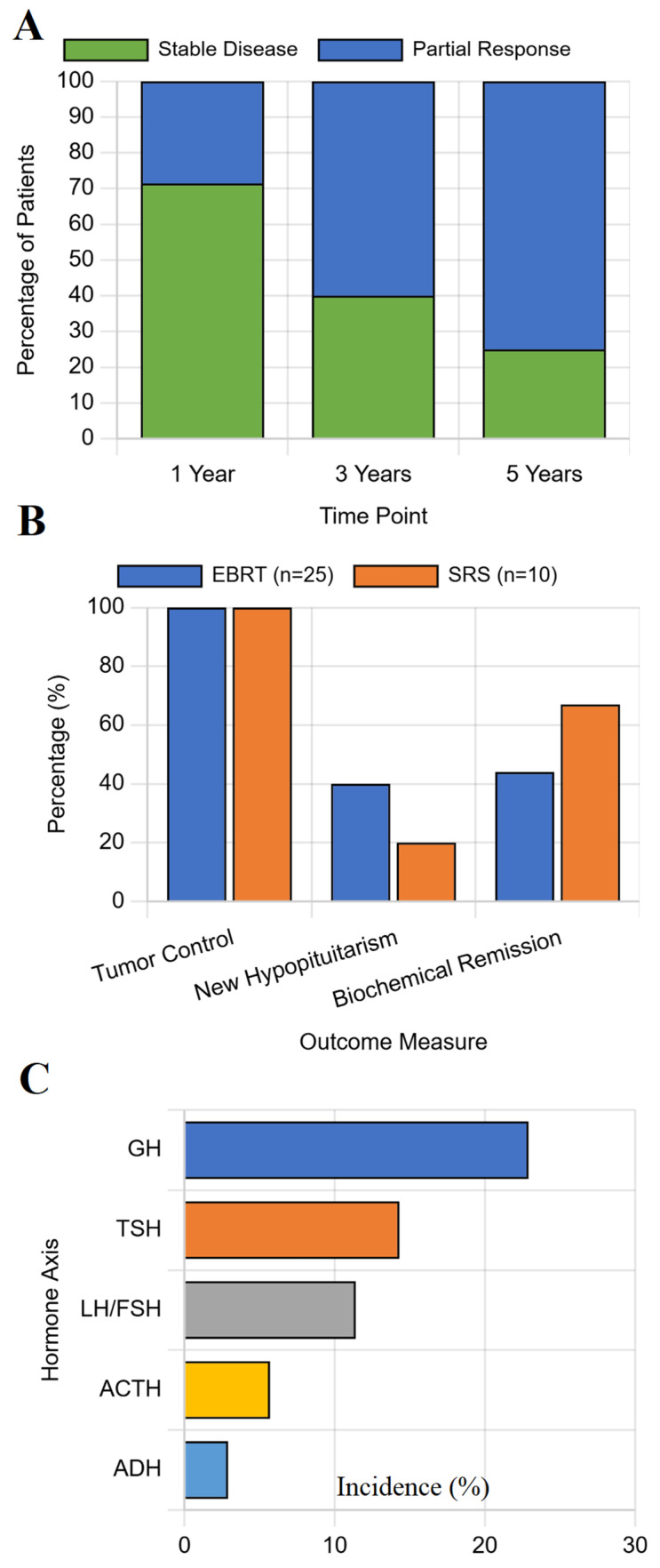
**Comparative outcomes by primary treatment modality.** Bar charts comparing 5-year outcomes. (**A**) Adenoma control rates. (**B**) Comparison of 5-year outcomes between EBRT (*n* = 25) and SRS (*n* = 10). (**C**) Incidence of new-onset hypopituitarism. Error bars represent 95% confidence intervals. Statistical comparisons performed using chi-square test. Abbreviations: ERBT, external beam radiotherapy; SRS, stereotactic radiosurgery; GH, growth hormone; TSH, thyroid-stimulating hormone; LH, luteinizing hormone; FSH, follicle-stimulating hormone; ACTH, adrenocorticotropic hormone; ADH, antidiuretic hormone (vasopressin).

With a median follow-up of 4.8 years (IQR 2.5–7.1 years), the overall adenoma control rate was 92.1% (198/215). Adenoma control was achieved in 100% of patients treated with radiotherapy, compared to 90.7% with medical therapy, 89.1% with surgery, and 86.7% with observation. While this trend towards superior control with radiotherapy was clinically meaningful, it did not reach statistical significance (*p* = 0.089), see Figure 2A for details.

Within the radiotherapy group, patients treated with EBRT had a significantly higher rate of new hypopituitarism compared to those treated with SRS (44.0% vs. 20.0%, *p* = 0.048), despite both modalities achieving 100% adenoma control, see Figure 2B.

The incidence of new-onset hypopituitarism varied dramatically by primary treatment modality (*p* < 0.001). The 5-year cumulative incidence was lowest for medical therapy (5.6%) and observation (6.7%), moderate for surgery (13.0%), and highest for radiotherapy (34.3%), see Figure 2C.

### 3.3. Predictors of Treatment-Related Hypopituitarism

In multivariable logistic regression analysis, several factors emerged as independent predictors of developing new-onset hypopituitarism (Table 2). The strongest predictor was treatment with radiotherapy (Odds Ratio 8.45, 95% Confidence Interval [CI]: 3.82–18.71, *p* < 0.001). The presence of pre-existing hypopituitarism at baseline also significantly increased the risk (OR 3.21, 95% CI: 1.58–6.52, *p* = 0.001). This strong predictive value suggests that patients with pre-existing hormonal deficits had diminished pituitary reserve,, rendering the remaining functional tissue more vulnerable to injury from subsequent treatment. Cavernous sinus invasion was another major predictor (OR 2.15, 95% CI: 1.03–4.49, *p* = 0.042). Macroadenoma and an age of >50 years showed trends towards significance and were retained in the final model for their clinical importance. See Table 2. The TRIPOD checklist for prediction model development can be seen in Supplementary Table S1.

### 3.4. Development of Model for Risk Prediction of Hypopituitarism 

**Figure 3:**
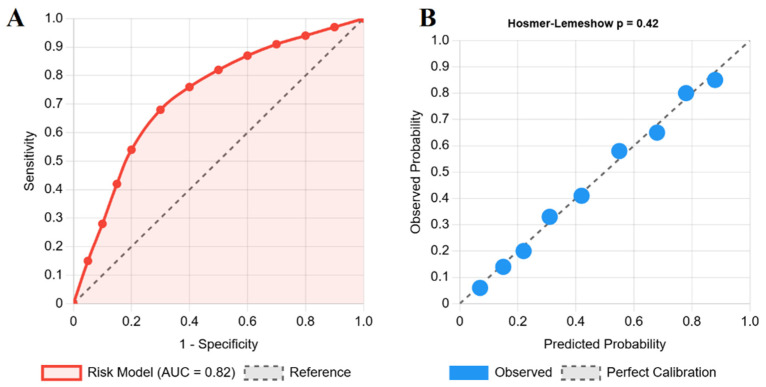
**Risk prediction model performance.** Evaluation of the final risk prediction model for new-onset hypopituitarism. (**A**) The Receiver Operating Characteristic (ROC) curve shows good discrimination, with an Area Under the Curve (AUC) of 0.82. (**B**) The calibration plot demonstrates high agreement between predicted probabilities and observed outcomes, supported by a non-significant Hosmer–Lemeshow test (*p* = 0.42).

The final risk prediction model included the following five variables: treatment modality, baseline hypopituitarism, adenoma size (macroadenoma), age (>50 years), and cavernous sinus invasion. The model demonstrated good discrimination, with a C-statistic of 0.82 (95% CI: 0.76–0.88), indicating a strong ability to distinguish between patients who did and did not develop hypopituitarism (Figure 3A). The model also showed excellent calibration, with high agreement between predicted probabilities and observed outcomes, confirmed by a non-significant Hosmer–Lemeshow test (*p* = 0.42) (Figure 3B).

Based on the regression coefficients, a simplified points-based risk score was created, as follows: Radiotherapy (4 points), Baseline hypopituitarism (2 points), Macroadenoma (2 points), Age > 50 years (1 point), and Cavernous sinus invasion (1 point). This scoring system stratified patients into the following three risk categories for developing new hypopituitarism: low risk (0–2 points, <10% probability), intermediate risk (3–5 points, 10–30% probability), and high risk (≥6 points, >30% probability). This scoring system was developed for clinical application [22], and is available as an online tool at https://hypopituitarism-risk.netlify.app/ (accessed on 13 September 2025).

**Figure 4:**
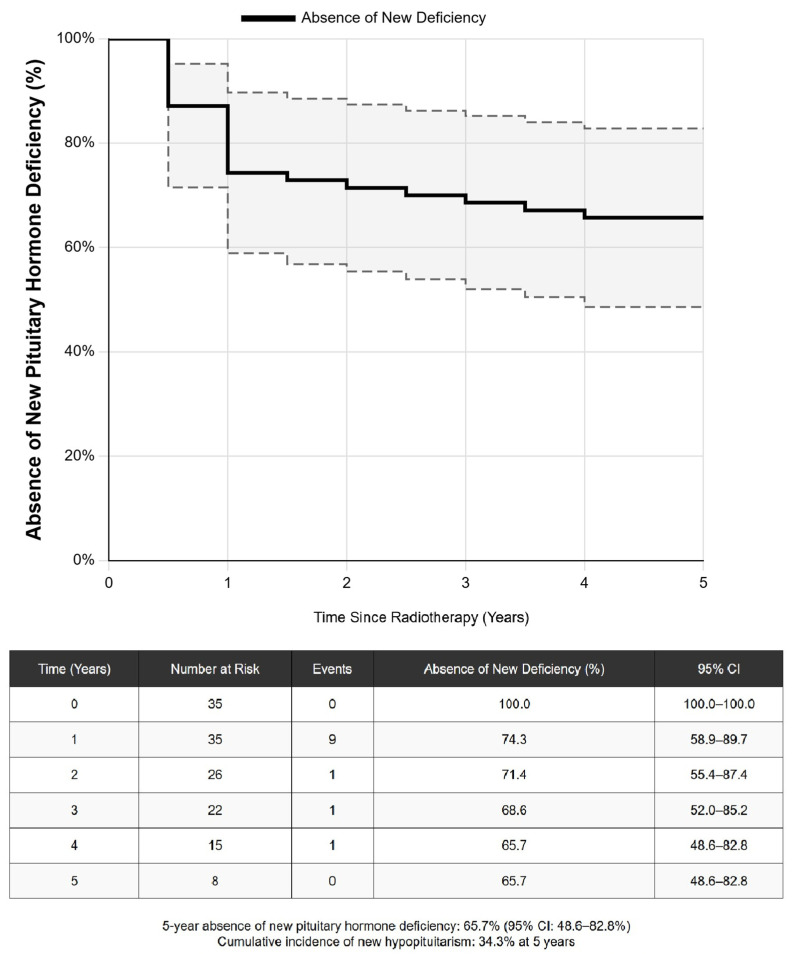
**Kaplan–Meier curve for absence of new hypopituitarism following radiotherapy (*N* = 35).** The survival curve illustrating the probability of remaining free from new hormone deficiencies over five years post-radiotherapy. The dotted line indicates 95% confidence interval. The table below the curve shows the number of patients at risk for developing new hormone deficiency at the beginning of each year.

Following radiotherapy, new pituitary hormone deficiencies developed in a time-dependent manner. The Kaplan–Meier estimated rates of freedom from new hypopituitarism were 74.3% at 1 year, 68.6% at 3 years, and 65.7% at 5 years, corresponding to a cumulative incidence of 34.3% at 5 years. See Figure 4 for details.

## 4. Discussion

This comprehensive analysis of 215 patients with pituitary adenomas provides a real-world perspective on treatment outcomes and culminates in the development of a novel, validated risk prediction model for treatment-related hypopituitarism. Our findings confirm the distinct risk–benefit profiles of modern therapeutic modalities and offer a pragmatic tool to guide individualized patient care.

### 4.1. Comparative Treatment Outcomes in a Real-World Setting

Our cohort’s demographic and adenoma distribution align with those of large epidemiological studies, enhancing the generalizability of our findings [1,2,23]. The treatment outcomes observed in our cohort from the United Arab Emirates provide a valuable benchmark against global standards. The 70.6% remission rate for prolactinomas treated with dopamine agonists is consistent with meta-analyses reporting 60–80% biochemical control [24,25]. Similarly, our overall surgical gross total resection rate of 48.9% reflects a real-world, unselected cohort that includes large and invasive adenomas, with outcomes for micro- and macroadenomas aligning with multicenter registries [26,27].

Radiotherapy demonstrated exceptional adenoma control (100% at 5 years), reinforcing its vital role for refractory or recurrent disease. This rate is at the high end of the 90–98% control rates reported in major radiotherapy series. However, this efficacy comes at a significant endocrine cost. The 34.3% cumulative incidence of new hypopituitarism at 5 years in our radiotherapy group falls within the 20–80% range reported in the literature but provides a precise, contemporary estimate. This rate is higher than the 13–21% incidence reported in some recent SRS-focused meta-analyses [28]. This difference is likely attributable to our inclusion of patients treated with EBRT, who experienced a significantly higher rate of hypopituitarism than SRS patients (44.0% vs. 20.0%). This finding underscores the importance of radiotherapy technique selection in mitigating long-term toxicity and is consistent with dosimetric studies showing superior sparing of normal tissue with SRS [29].

### 4.2. A Pragmatic Tool for Individualized Risk Prediction

The principal contribution of this study is the development and validation of a clinical risk score for new-onset hypopituitarism. While the individual predictors identified—radiotherapy, baseline hypopituitarism, and features of tumor aggressiveness—are not surprising, the novelty of our model lies in its ability to integrate these factors into a single, quantitative, and transparent score. The model’s strong performance (C-statistic = 0.82) provides a validated tool that transforms a qualitative understanding of risk into a personalized, quantitative estimate that can directly inform shared decision making. For a patient with a recurrent, invasive macroadenoma and pre-existing gonadotropin deficiency, our model can quantify the risk of further pituitary damage with radiotherapy versus repeat surgery, allowing for a more informed discussion of treatment options.

The clinical utility of this model extends beyond treatment selection to inform post-treatment surveillance. For instance, a patient classified as high-risk (≥6 points; >30% probability of hypopituitarism) warrants more intensive monitoring, such as annual comprehensive pituitary function testing for at least the first five years post-treatment. Conversely, a low-risk patient might be monitored less frequently. This risk-stratified approach can optimize healthcare resource allocation while ensuring patient safety. This pragmatic approach offers an accessible and transparent alternative to more complex AI or ML models, which, despite their potential for high accuracy, often lack the interpretability required for routine clinical adoption [30].

While individual risk factors for hypopituitarism are well-recognized, our study’s principal innovation lies in integrating these factors into a validated, quantitative scoring system. This transforms qualitative clinical intuition into an actionable, evidence-based tool that can standardize risk assessment across different centers and experience levels.

### 4.3. Strengths and Limitations

The strengths of this study include its comprehensive long-term follow-up, systematic endocrine and radiological monitoring, the inclusion of all adenoma subtypes and treatment modalities, and the inclusion of a large, diverse cohort from the Middle East, a region underrepresented in the pituitary literature, which enhances the global applicability of our findings. The development of a clinically applicable and internally validated risk model is a primary strength.

However, several limitations must be acknowledged. The retrospective design is susceptible to selection bias, although we mitigated this by including a consecutive series of patients. The single-center nature of the study may limit its generalizability, and external validation of the risk model in a separate, multi-center cohort is a crucial next step before widespread implementation. Furthermore, our analysis did not include detailed dosimetric data for radiotherapy patients, such as the mean dose to the pituitary gland or hypothalamus, which have been identified as important predictors of hypopituitarism in recent studies [31]. Finally, the relatively small number of patients in certain subgroups, particularly the SRS group, limited the statistical power for some sub-analyses.

## 5. Conclusions

In this large cohort analysis, medical, surgical, and radiotherapeutic modalities for pituitary adenomas demonstrated distinct efficacy and morbidity profiles. Medical therapy for prolactinomas and surgery for other adenomas remain effective primary treatments with favorable risk profiles. Radiotherapy provides exceptional long-term adenoma control for refractory or recurrent disease but at the substantial cost of new-onset hypopituitarism, which develops in over one-third of patients within five years.

Our validated, points-based risk score is the first to provide a simple, quantitative tool to predict the individualized risk of treatment-related hypopituitarism. This model can be readily integrated into clinical workflows to enhance shared decision making, guide treatment selection, and tailor post-treatment surveillance strategies, thereby advancing a more personalized approach to pituitary adenoma management.

Future research should focus on the external validation of this risk model in multicenter, prospective cohorts. Furthermore, head-to-head comparisons with emerging machine learning algorithms are warranted to determine the optimal balance between predictive accuracy and clinical interpretability. Finally, studies exploring novel radioprotective strategies and advanced radiotherapy techniques, such as proton therapy, are crucial to mitigate the significant risk of endocrine dysfunction highlighted in our study.

## Figures and Tables

**Table 1:** Baseline characteristics of patients with pituitary adenoma.

Characteristics	Total Cohort (*N* = 215)	Radiotherapy Group (*N* = 35)	No Radiotherapy (*N* = 180)	*p*-Value
Demographics				
Age at diagnosis, mean ± SD	43.2 ± 14.1	48.1 ± 12.3	42.2 ± 14.3	0.018
Female, *n* (%)	127 (59.1)	18 (51.4)	109 (60.6)	0.312
BMI, mean ± SD	27.8 ± 5.2	28.4 ± 4.9	27.7 ± 5.3	0.462
Adenoma type, *n* (%)				
Prolactinoma	107 (49.8)	5 (14.3)	102 (56.7)	<0.001
Non-functioning	77 (35.8)	20 (57.1)	57 (31.7)	
GH-secreting	18 (8.4)	7 (20.0)	11 (6.1)	
ACTH-secreting	8 (3.7)	2 (5.7)	6 (3.3)	
Other/Multiple	5 (2.3)	1 (2.9)	4 (2.2)	
Adenoma size characteristics				
Macroadenoma, *n* (%)	117 (54.4)	29 (82.9)	88 (48.9)	<0.001
Maximum diameter, mm	18.2 ± 11.4	26.8 ± 12.1	16.5 ± 10.3	<0.001
Cavernous sinus invasion, *n* (%)	45 (20.9)	15 (42.9)	30 (16.7)	<0.001
Suprasellar extension, *n* (%)	89 (41.4)	24 (68.6)	65 (36.1)	<0.001
Clinical presentation				
Headache, *n* (%)	102 (47.4)	22 (62.9)	80 (44.4)	0.044
Visual field defect, *n* (%)	48 (22.3)	15 (42.9)	33 (18.3)	0.002
Incidental finding, *n* (%)	28 (13.0)	2 (5.7)	26 (14.4)	0.159
Baseline hormonal status				
Any hypopituitarism, *n* (%)	71 (33.0)	19 (54.3)	52 (28.9)	0.004
Number of deficient axes	0.6 ± 1.0	1.1 ± 1.3	0.5 ± 0.9	0.001
GH deficiency, *n* (%)	32 (14.9)	10 (28.6)	22 (12.2)	0.014
TSH deficiency, *n* (%)	28 (13.0)	8 (22.9)	20 (11.1)	0.058
ACTH deficiency, *n* (%)	21 (9.8)	5 (14.3)	16 (8.9)	0.323
LH/FSH deficiency, *n* (%)	43 (20.0)	12 (34.3)	31 (17.2)	0.023
Panhypopituitarism, *n* (%)	11 (5.1)	4 (11.4)	7 (3.9)	0.071

Comparison of demographic, clinical, and adenoma characteristics between the total patient cohort (*N* = 215) and the subgroup that received radiotherapy (*N* = 35). Continuous variables were analyzed with Student’s *t*-test; categorical variables with chi-square or Fisher’s exact test. Abbreviations: BMI, body mass index; GH, growth hormone; TSH, thyroid-stimulating hormone; ACTH, adrenocorticotropic hormone; LH, luteinizing hormone; FSH, follicle-stimulating hormone. Number of deficient axes is presented as median due to non-normal distribution.

**Table 2:** Multivariable analysis of predictors for new-onset hypopituitarism.

Variable	Univariable Analysis OR (95% CI)	*p*-Value	Multivariable Analysis OR (95% CI)	*p*-Value
Treatment Modality				
Medical therapy	Reference		Reference	
Surgery	2.52 (0.94–6.75)	0.066	2.18 (0.78–6.09)	0.138
Radiotherapy	9.21 (3.35–25.32)	<0.001	8.45 (3.82–18.71)	**<0.001**
Observation	0.89 (0.18–4.42)	0.887	0.92 (0.17–4.98)	0.923
Baseline Characteristics				
Age > 50 years	2.31 (1.21–4.41)	0.011	1.92 (0.96–3.84)	0.065
Adenoma Characteristics				
Macroadenoma	3.45 (1.52–7.83)	0.003	2.01 (0.82–4.93)	0.127
Cavernous sinus invasion	2.78 (1.44–5.37)	0.002	2.15 (1.03–4.49)	**0.042**
Suprasellar extension	1.92 (1.01–3.65)	0.047	1.45 (0.71–2.96)	0.308
Baseline Hormonal Status				
Pre-existing hypopituitarism	4.12 (2.13–7.97)	<0.001	3.21 (1.58–6.52)	**0.001**
Radiotherapy-Specific (*n* = 35)				
EBRT vs. SRS	3.14 (1.12–8.81)	0.030	2.78 (1.15–6.72)	**0.023**
Dose > 50 Gy	2.89 (1.23–6.79)	0.015	2.45 (0.98–6.13)	0.055
Previous surgery before RT	1.67 (0.48–5.81)	0.421		

Univariable and multivariable logistic regression analysis identifying predictors for the development of new-onset hypopituitarism. **Bold values** indicate statistical significance (*p* < 0.05) in the final multivariable model. Model Performance: C-statistic = 0.82 (95% CI: 0.76–0.88); Hosmer–Lemeshow goodness-of-fit test *p* = 0.42. Abbreviations: OR, odds ratio; CI, confidence interval; EBRT, external beam radiotherapy; SRS, stereotactic radiosurgery; RT, radiotherapy; Gy, Gray.

## Data Availability

The raw data supporting the conclusions of this article will be made available by the authors on request.

## Supplementary Materials

The following supporting information can be downloaded at: https://www.mdpi.com/article/10.3390/jcm14186656/s1 Supplementary Table S1, The TRIPOD checklist for prediction model development.

## References

[1] Ezzat S., Asa S.L., Couldwell W.T., Barr C.E., Dodge W.E., Vance M.L., McCutcheon I.E. (2004). The prevalence of pituitary adenomas: A systematic review. Cancer.

[2] Fernandez A., Karavitaki N., Wass J.A. (2010). Prevalence of pituitary adenomas: A community-based, cross-sectional study in Banbury (Oxfordshire, UK). Clin. Endocrinol..

[3] Melmed S. (2020). Pituitary-adenoma endocrinopathies. N. Engl. J. Med..

[4] Fleseriu M., Hashim I.A., Karavitaki N., Melmed S., Murad M.H., Salvatori R., Samuels M.H. (2016). Hormonal replacement in hypopituitarism in adults: An Endocrine Society clinical practice guideline. J. Clin. Endocrinol. Metab..

[5] Molitch M.E. (2017). Diagnosis and treatment of pituitary adenomas: A review. JAMA.

[6] Hall W.A., Luciano M.G., Doppman J.L., Patronas N.J., Oldfield E.H. (1994). Pituitary magnetic resonance imaging in normal human volunteers: Occult adenomas in the general population. Ann. Intern. Med..

[7] Petersenn S., Fleseriu M., Casanueva F.F., Giustina A., Biermasz N., Biller B.M.K., Bronstein M., Chanson P., Fukuoka H., Gadelha M. (2023). Diagnosis and management of prolactin-secreting pituitary adenomas: A Pituitary Society international consensus statement. Nat. Rev. Endocrinol..

[8] Zaidi H.A., Awad A.W., Bohl M.A., Chapple K., Knecht L., Jahnke H., White W.L., Little A.S. (2016). Comparison of outcomes between a less experienced surgeon using a fully endoscopic technique and a very experienced surgeon using a microscopic transsphenoidal technique for pituitary adenoma. J. Neurosurg..

[9] Minniti G., Flickinger J., Tolu B., Paolini S. (2023). Radiotherapy and radiosurgery for pituitary adenomas. Pituitary.

[10] Sheehan J.P., Pouratian N., Steiner L., Laws E.R., Vance M.L. (2011). Gamma Knife surgery for pituitary adenomas: Factors related to radiological and endocrine outcomes. J. Neurosurg..

[11] Tomlinson J.W., Holden N., Hills R.K., Wheatley K., Clayton R.N., Bates A., Sheppard M., Stewart P. (2001). Association between premature mortality and hypopituitarism. Lancet.

[12] Higham C.E., Johannsson G., Shalet S.M. (2016). Hypopituitarism. Lancet.

[13] Xu Z., Vance M.L., Schlesinger D., Sheehan J.P. (2013). Hypopituitarism after stereotactic radiosurgery for pituitary adenomas. Neurosurgery.

[14] Appelman-Dijkstra N.M., Malgo F., Neelis K.J., Coremans I., Biermasz N.R., Pereira A.M. (2014). Pituitary dysfunction in adult patients after cranial irradiation for head and nasopharyngeal tumours. Radiother. Oncol..

[15] Cordeiro D., Xu Z., Mehta G.U., Ding D., Vance M.L., Kano H., Sisterson N., Yang H.C., Kondziolka D., Lunsford L.D. (2018). Hypopituitarism after Gamma Knife radiosurgery for pituitary adenomas: A multicenter, international study. J. Neurosurg..

[16] Mohammadzadeh I., Hajikarimloo B., Niroomand B., Eini P., Habibi M.A., Mortezaei A., Bagheri M.H., Günkan A., Aaronson D.M., Himic V. (2025). Using machine learning to predict remission after surgery for pituitary adenoma: A systematic review and meta-analysis. Endocrine.

[17] Fleseriu M., Gurnell M., McCormack A., Fukuoka H., Glezer A., Langlois F., Schwartz T.H., Greenman Y., Agrawal N., Akirov A. (2025). for The Pituitary Society International Incidentaloma Consensus Group. Pituitary incidentaloma: A Pituitary Society international consensus guideline statement. Nat. Rev. Endocrinol..

[18] Nieman L.K., Biller B.M., Findling J.W., Newell-Price J., Savage M.O., Stewart P.M., Montori V.M. (2008). The diagnosis of Cushing’s syndrome: An Endocrine Society Clinical Practice Guideline. J. Clin. Endocrinol. Metab..

[19] Katznelson L., Laws E.R., Melmed S., Molitch M.E., Murad M.H., Utz A., Wass J.A.H. (2014). Acromegaly: An endocrine society clinical practice guideline. J. Clin. Endocrinol. Metab..

[20] Van den Bergh A.C.M., Van den Berg G.A., Schoorl M.A., Sluiter W.J., van der Vliet A.M., Hoving E.W., Szabó B.G., Langendijk J.A., Wolffenbuttel B.H., Dullaart R.P. (2020). Immediate postoperative radiotherapy in residual nonfunctioning pituitary adenoma: Beneficial effect on local control without additional negative impact on pituitary function and life expectancy. Int. J. Radiat. Oncol. Biol. Phys..

[21] Chen Y., Li Z.F., Zhang F.X., Li J.X., Cai L., Zhuge Q.C., Wu Z.B. (2013). Gamma knife surgery for patients with volumetric classification of nonfunctioning pituitary adenomas: A systematic review and meta-analysis. Eur. J. Endocrinol..

[22] Agha A. Pituitary Adenoma Hypopituitarism Risk Calculator. https://hypopituitarism-risk.netlify.app/.

[23] Dekkers O.M., Hammer S., de Keizer R.J., Roelfsema F., Schutte P.J., Smit J.W.A., Romijn J.A., Pereira A.M. (2007). The natural course of non-functioning pituitary macroadenomas. Eur. J. Endocrinol..

[24] Dos Santos Nunes V., El Dib R., Boguszewski C.L., Nogueira C.R. (2011). Cabergoline versus bromocriptine in the treatment of hyperprolactinemia: A systematic review of randomized controlled trials and meta-analysis. Pituitary.

[25] Molitch M.E. (2014). Management of medically refractory prolactinoma. J. Neurooncol..

[26] Ammirati M., Wei L., Ciric I. (2013). Short-term outcome of endoscopic versus microscopic pituitary adenoma surgery: A systematic review and meta-analysis. J. Neurol. Neurosurg. Psychiatry.

[27] Tabaee A., Anand V.K., Barrón Y., Hiltzik D.H., Brown S.M., Kacker A., Mazumdar M., Schwartz T.H. (2009). Endoscopic pituitary surgery: A systematic review and meta-analysis. J. Neurosurg..

[28] Pollock B.E., Cochran J., Natt N., Brown P.D., Erickson D., Link M.J., Garces Y.I., Foote R.L., Stafford S.L., Schomberg P.J. (2008). Gamma knife radiosurgery for patients with nonfunctioning pituitary adenomas: Results from a 15-year experience. Int. J. Radiat. Oncol. Biol. Phys..

[29] McLaren D.S., Devi A., Kyriakakis N., Kwok-Williams M., Murray R.D. (2023). The impact of radiotherapy on the hypothalamo-pituitary axis: Old vs new radiotherapy techniques. Endocr. Connect..

[30] Zhang J., Zhang Z.M. (2023). Ethics and governance of trustworthy medical artificial intelligence. BMC Med. Inform. Decis. Mak..

[31] Kyriakakis N., Lynch J., Orme S.M., Gerrard G., Hatfield P., Short S.C., Loughrey C., Murray R.D. (2019). Hypothalamic-pituitary axis irradiation dose thresholds for the development of hypopituitarism in adult-onset gliomas. Clin. Endocrinol..

