# Supplementary material for: Risk-Stratifying Pituitary Adenoma Treatment: A Cohort Analysis and Risk Prediction of Hypopituitarism
Source: J Clin Med. 2025 Sep 22;14(18):6656. doi: 10.3390/jcm14186656 (PMC12471088; doi:10.3390/jcm14186656)
Supplement: Supplementary file 1 [file jcm-14-06656-s001.zip › jcm-3846747-supplementary.pdf]

TRIPOD Checklist: Prediction Model Development

Study: Risk-Stratifying Pituitary Adenoma Treatment: A Cohort Analysis and Predicting Risk of Hypopituitarism

Section/Topic	Item	Checklist Item	Page/Location
**TITLE AND ABSTRACT**			
Title	1	Identify the study as developing and/or validating a multivariable prediction model, the target population, and the outcome to be predicted	Title page - "Predicting Risk of Hypopituitarism"
Abstract	2	Provide a summary of objectives, study design, setting, participants, sample size, predictors, outcome, statistical analysis, results, and conclusions	Abstract - all elements included
**INTRODUCTION**			
Background and objectives	3a	Explain the medical context (including whether diagnostic or prognostic) and rationale for developing or validating the multivariable prediction model	Introduction, paragraphs 3-4
	3b	Specify the objectives, including whether the study describes the development or validation of the model or both	Introduction, final paragraph - "develop a clinically applicable risk prediction model"
**METHODS**			
Source of data	4a	Describe the study design or source of data (e.g., randomized trial, cohort, or registry data), separately for the development and validation data sets, if applicable	Methods, section 1 - "retrospective cohort study"
	4b	Specify the key study dates, including start of accrual; end of accrual; and, if applicable, end of follow-up	Methods, section 1 - "January 2010 and December 2020"
Participants	5a	Specify key elements of the study setting (e.g., primary care, secondary care, general population) including number and location of centres	Methods, section 1 - "Tawam Hospital, Al-Ain United Arab Emirates"
	5b	Describe eligibility criteria for participants	Methods, Selection criteria
	5c	Give details of treatments received, if relevant	Methods, Treatment Protocols

Section/Topic	Item	Checklist Item	Page/Location
Outcome	6a	Clearly define the outcome that is predicted by the prediction model, including how and when assessed	Methods, Outcome Measures - "New hypopituitarism"
	6b	Report any actions to blind assessment of the outcome to be predicted	N/A - retrospective study
Predictors	7a	Clearly define all predictors used in developing or validating the multivariable prediction model, including how and when they were measured	Methods, Clinical Assessment and Treatment Protocols
	7b	Report any actions to blind assessment of predictors for the outcome and other predictors	N/A - retrospective study
Sample size	8	Explain how the study size was arrived at	Methods, section 1 - consecutive sampling
Missing data	9	Describe how missing data were handled (e.g., complete-case analysis, imputation of missing values) with details of any imputation method	Methods, Statistical Analysis
Statistical analysis methods	10a	Describe how predictors were handled in the analyses	Methods, Statistical Analysis
	10b	Specify type of model, all model-building procedures (including any predictor selection), and method for internal validation	Methods, Risk Prediction Model Development
	10c	For validation, describe how the predictions were calculated	Methods, Risk Prediction Model Development
	10d	Specify all measures used to assess model performance and, if relevant, to compare multiple models	Methods, Risk Prediction Model Development - "C-statistic and calibration using Hosmer-Lemeshow test"
	10e	Describe any model updating (from external validation) conducted	N/A - development only
Risk groups	11	Provide details on how risk groups were created, if done	Results, section 5 - "Risk scores ranged from 0-10 points"
**RESULTS**			
Participants	13a	Describe the flow of participants through the study, including the number of participants with and without the outcome and, if applicable, a summary of the follow-up time	Figure 1, Results section 1

Section/Topic	Item	Checklist Item	Page/Location
	13b	Describe the characteristics of the participants (basic demographics, clinical features, available predictors), including the number of participants with missing data for predictors and outcome	Table 1, Results section 1
	13c	For validation, show a comparison with the development data set of the distribution of important variables (demographics, predictors and outcome)	N/A - development only
Model development	14a	Specify the number of participants and outcome events in each analysis	Results, section 4 - "215 patients"
	14b	If done, report the unadjusted association between each candidate predictor and outcome	Table 2 - univariable analysis
Model specification	15a	Present the full prediction model to allow predictions for individuals (i.e., all regression coefficients, and model intercept or baseline survival at a given time point)	Results, section 5 - Risk Prediction Model
	15b	Explain how to use the prediction model	Results, section 5; Discussion, Clinical Implementation
Model performance	16	Report performance measures (with CIs) for the prediction model	Results, section 5 - "C-statistic=0.82"
Model-updating	17	If done, report the results from any model updating (i.e., model specification, model performance)	N/A
**DISCUSSION**			
Limitations	18	Discuss any limitations of the study (such as nonrepresentative sample, few events per predictor, missing data)	Discussion, Study Strengths and Limitations
Interpretation	19a	For validation, discuss the results with reference to performance in the development data set, and any other validation data sets	N/A - development only
	19b	Give an overall interpretation of the results, considering objectives, limitations, results from similar studies, and other relevant evidence	Discussion, sections 1-5
Implications	20	Discuss the potential clinical use of the model and implications for future research	Discussion, Clinical Implementation and Future Directions

Section/Topic	Item	Checklist Item	Page/Location
**OTHER INFORMATION**			
Supplementary information	21	Provide information about the availability of supplementary resources, such as study protocol, Web calculator, and data sets	Title page, Data Availability
Funding	22	Give the source of funding and the role of the funders for the present study	Title page, Declarations

TRIPOD Compliance Statement:

This manuscript adheres to the TRIPOD (Transparent Reporting of a multivariable prediction model for Individual Prognosis Or Diagnosis) Statement for reporting prediction model development. The risk prediction model for treatment-related hypopituitarism is clearly described with all predictors specified (treatment modality, baseline hypopituitarism, tumor size, age, and cavernous sinus invasion), model performance metrics reported (C-statistic = 0.82, calibration plot provided), and clinical implementation guidance included. The model development process, from predictor selection through internal validation, is transparently documented. Risk categories are defined with corresponding predicted probabilities to facilitate clinical use.
